# Health inequality and improvement gap in the prevalence of gynecological cancers among perimenopausal women globally, 1990–2019

**DOI:** 10.1186/s12889-025-21807-3

**Published:** 2025-02-12

**Authors:** Chenglin Yang, Jianqin Zou, Xiaochen Luo, Yangjiang Ou, Xiaoru Lin, Xinyu Wang, Qing Guan, Fanxiang Zeng, Dong Liang, Xiuquan Lin

**Affiliations:** 1 Department for Chronic and Noncommunicable Disease Control and Prevention, Fujian Provincial Center for Disease Control and Prevention, No. 386 Chong ’an Road, Jin’an District, Fuzhou, Fujian China; 2 The School of Public Health, Fujian Medical University, Fuzhou, Fujian China; 3 Department of Obstetrics and Gynecology, Affiliated Fuzhou First General Hospital of Fujian Medical University, Fujian, China; 4 The School of Health Management, Fujian Medical University, Fuzhou, China Fujian; 5 The 14th Five-Year Plan” Application Characteristic Discipline of Hunan Province (Clinical Medicine), Changsha Medical University, Changsha, Hunan China; 6 The School of Public Health, Xiamen University, Xiamen, Fujian China; 7 Department of Society and Health, Faculty of Social Sciences and Humanities, Mahidol University, Nakhon Pathom, Thailand; 8 Fujian Maternity and Child Health Hospital, No. 18 Daoshan Road, Gulou District, Fuzhou, Fujian China; 9 Fujian Key Laboratory of Women and Children’s Critical Diseases Research [Fujian Maternity and Child Health, Hospital(Fujian Women and Children’s Hospital)], Fuzhou, Fujian China

**Keywords:** Gynecological cancer, Health inequality, Perimenopausal women, Frontier analysis

## Abstract

**Background:**

Perimenopausal women are a high-risk group for gynecological cancers; however, the spatiotemporal heterogeneity in prevalence and its association with socioeconomic development have not been fully explored. This study aimed to analyze the global burden of cervical, ovarian, and uterine cancers among perimenopausal women, examine health inequalities, and investigate their relationship with socioeconomic levels.

**Methods:**

We assessed the disease burden of gynecological cancers in perimenopausal women using the 2019 Global Burden of Disease study (GBD2019) data, utilizing prevalence and Years of Life Lost (YLL) rates. Health inequality and frontier analyses were performed based on age-standardized prevalence rates for cervical, ovarian, and uterine cancers, coupled with associated socio-demographic index (SDI) data.

**Results:**

Over the past thirty years, global prevalence rates of cervical, ovarian, and uterine cancers among perimenopausal women have risen, while the number of YLLs has declined. Correlation analysis with the SDI showed that ovarian and uterine cancer prevalence rates positively correlated with SDI, whereas cervical cancer prevalence was inversely related to it. Moreover, the burden of these cancers demonstrated marked inequalities in relation to SDI, with cervical cancer disparities intensifying—the absolute value of the inequality slope index rose from 100.98 in 1990 to 170.17 in 2019. Ovarian cancer experienced a slight decrease in inequalities, while uterine cancer saw a significant increase, with its inequality slope index jumping from 176.51 in 1990 to 226.01 in 2019. Additionally, there existed regional health disparities in the disease burden of ovarian cancer among perimenopausal women; notably, in regions where YLLs rates for ovarian cancer were increasing, economically developed areas actually exhibited a decline.

**Conclusion:**

Globally, the prevalence of gynecological cancers in perimenopausal women showed an increasing trend. The health inequality gap for cervical and uterine cancer has widened, while disparities in ovarian cancer are particularly pronounced across regions. There remains scope for improvement in managing the prevalence of gynecological cancers among perimenopausal women across countries with varying SDI levels.

**Supplementary Information:**

The online version contains supplementary material available at 10.1186/s12889-025-21807-3.

## Introduction

Perimenopause referred to the transitional period from the onset of ovarian dysfunction to menopause, lasting until one year after a woman’s final menstrual period [1]. Perimenopause was typically associated with changes in the reproductive system [2]. Cancers of the female reproductive system are important contributors to global cancer morbidity and mortality, with higher incidence of ovarian, uterine, and cervical cancers [3]; some of these cancers are hormone-related [4–7]. During perimenopause, obesity is a significant issue faced by women [8]. Furthermore, studies have shown that obesity is a key risk factor for uterine cancer [9], increasing the risk of uterine cancer in perimenopausal women. Another study indicates that the burden of ovarian cancer is heaviest in individuals aged 50 and older [10].Furthermore, in Africa, the Americas, and Europe, a clear second peak of HPV prevalence was observed in women aged 45 years or older, and most cases of cervical cancer occur as a result of infections with HPV types 16 and 18 [11, 12]. Based on the above content, perimenopausal women are a high-risk group for developing gynecological cancers. Therefore, understanding the prevalence of gynecological cancers in perimenopausal women is particularly crucial.

Survival rates vary among different gynecological cancers. The five-year relative survival rate is 50.9% for patients with ovarian cancer [13], 67% for cervical cancer patients [14], and higher for uterine cancer patients, with a five-year relative survival rate of 80.8% [15]. Perimenopausal women are prone to experiencing issues such as palpitations, insomnia, depression, and anxiety [16], all of which may potentially affect cancer treatment. Additionally, significant socioeconomic disparities exist in female cancers, presenting notable health challenges and burdens across regions and countries with varying economic statuses [17, 18].

Currently, there are disparities in the regional and national accessibility to medical care for reproductive cancer [19–21]. While many scholars have shown a keen interest in gynecological cancers, there is a lack of global overviews and analyses concerning the prevalence rates of gynecological cancers in perimenopausal women. Therefore, estimating age-standardized prevalence rates of gynecological cancers in perimenopausal women globally and exploring their relation with socioeconomic development can address deficiencies in disease statistics. This effort can contribute to a better understanding of disease epidemiology, assist in addressing health inequality issues related to such diseases across nations, promote relevant medical practices, seek solutions to alleviate the disease burden of gynecological cancers in perimenopausal women and advance global discussions on perimenopausal women’s health.

This study utilized data from the 2019 Global Burden of Disease study(GBD 2019)to estimate the prevalence rates and years of life lost (YLL) due to gynecological cancers in perimenopausal women from 1990 to 2019, including ovarian cancer, uterine cancer, and cervical cancer. Additionally, this study further analyzed the global, regional, and national trends of these diseases over the past three decades, examining their relation with economic development and the impact of cross-national inequalities. These findings enhance our understanding of the significance of gynecological cancer prevalence among perimenopausal women and contribute to advancing international efforts to improve screening methods for gynecological cancers.

## Methods

### Overview

The methods related to computing data for GBD 2019 are documented in published articles [22, 23]. GBD 2019 employs various metrics to assess population health loss, including incidence and mortality rates, case counts and prevalence, YLLs, years lived with disability (YLDs), and disability adjusted life years (DALYs). In the GBD 2019 study, the socio-demographic index (SDI) was calculated based on the fertility rate of young women, the educational attainment of women aged 15 or older, and per capita income adjusted for distribution. SDI ranges from 0 to 1, where 0 represents minimal education, lowest income, and highest fertility rates, and vice versa. According to the GBD 2019 study, the SDIs were sorted into five categories: high SDI (greater than 0.81), high-middle SDI (0.70–0.81), middle SDI (0.61–0.69), low-middle SDI (0.46–0.60), and low SDI (less than 0.46) The above information is all available online for public access at https://vizhub.healthdata.org/gbd-results. This study extracted and analyzed global data on gynecological cancers in perimenopausal women from 1990 to 2019.

### Indicator extraction

The World Health Organization (WHO) monograph “Research on the Menopause in the 1990s” formulates the most appropriate definition of the perimenopause. “The term perimenopause should include the period immediately before the menopause (when the endocrinological, biological and clinical features of approaching menopause commence) and the first year after menopause [1]. The American College of Obstetricians and Gynecologists (ACOG) indicated that while the duration of perimenopause varied from person to person, most perimenopausal periods occurred between the ages of 45 and 55 [24]. We extracted data from the GBD database for women aged 45–54 years with gynecological cancers (cervical cancer, ovarian cancer, and uterine cancer) from 1990 to 2019, including the number of cases, age-standardized prevalence rates, and YLLs rates. The detailed correspondence between cervical cancer, ovarian cancer, uterine cancer, and the International Classification of Diseases 10th Edition (ICD-10) codes can be found in Appendix Table S9.

### Statistical analysis

The data extracted was organized and analyzed to describe the distribution of age-standardized prevalence rates and YLLs rates for gynecological cancers (ovarian, uterine and cervical cancers) in perimenopausal women at the global, regional, and country-levels along with their 95% uncertainty intervals (UI). To understand trends in prevalence and YLLs rates burden, the average annual percentage change (AAPC) trends of age-standardized rates were calculated using a joinpoint regression method along with their significance. The relationship between disease prevalence in 204 countries and 21 regions and the SDI was studied using a generalized additive model (GAM). GAM is a sum of smooth functions that is suitable for analyzing various distribution types as well as complex nonlinear relationships. By modeling with smooth splines, it achieves high levels of fit while also providing smooth curves [25].

To further quantify inequalities in the burden of gynecological cancers among perimenopausal women related to SDI in different countries, slope and concentration indices were used to examine absolute and relative inequalities. The slope inequality index provides two standard measures for absolute and relative gradient inequalities, quantifying the distribution of disease burden across countries among perimenopausal women [26]. The slope inequality index regresses country prevalence rates of perimenopausal women on a relative positional scale associated with the SDI, defined by the midpoint of cumulative population ranges ranked by SDI. Heteroscedasticity was addressed using a weighted regression model. The concentration index calculates the area under the Lorenz concentration curve by numerical integration, fitting the cumulative portion of prevalence rates and the cumulative relative distribution of populations ranked by SDI.

In addition, to assess the relationship between the burden of gynecological cancers in perimenopausal women and population development, we employed frontier analysis as a quantitative approach to identify the potential minimum burden of cancer. Analysis was conducted using statistical software the R version 4.2.1.

## Results

### The prevalence and YLLs rates of gynecological cancers at the global, regional, and national levels

#### Cervical cancer

From to 2019, the global prevalence of cervical cancer among perimenopausal women aged 45–54 showed an increasing trend [45–49 years: 0.27% (95% CI: 0.01–0.54%); 50–54 years: 0.32% (95% CI: 0.11–0.53%)] (Appendix table S1). Over the same period, the global YLLs rates for cervical cancer in this group exhibited a decreasing trend [45–49 years: -0.99% (95% CI: -1.13% to -0.86%); 50–54 years: -0.86% (95% CI: -1.21% to -0.51%)] (Appendix table S2).

Between and 2019, the prevalence of cervical cancer declined in most regions, with Australasia experiencing the largest decrease [45–49 years: -1.25% (95% CI: -1.53% to -0.97%); 50–54 years: -1.22% (95% CI: -1.71% to -0.72%)] (Appendix table S1). Similarly, YLLs rates decreased across most regions, with Australasia showing the steepest decline [45–49 years: -2.65% (95% CI: -2.86% to -2.43%); 50–54 years: -2.33% (95% CI: -2.67% to -2.00%)] (Appendix table S2)

At the national level in 2019, the highest prevalence of cervical cancer (per 100,women) was recorded in Kiribati [45–49 years: 1,102.82 (95% CI: 645.27–1,712.45); 50–54 years: 1,007.31 (95% CI: 641.90–1,559.87)], while the lowest was observed in Egypt [45–49 years: 23.23 (95% CI: 12.92–37.96); 50–54 years: 25.34 (95% CI: 12.71–42.89)] (Appendix table S3). The age-standardized prevalence rates of cervical cancer for women aged 45–54 in 2019 were illustrated on the map (Appendix figure S1). From 1990 to 2019, the percentage change in prevalence varied by country, with 45 countries showing an upward trend for women aged 45–49 and 50 countries for women aged 50–54 (Appendix table S3)

#### Ovarian cancer

From 1990 to 2019, the global prevalence of ovarian cancer among perimenopausal women showed an increasing trend [45–49 years: 0.55% (95% CI: 0.40–0.71%); 50–54 years: 0.33% (95% CI: 0.16–0.50%)] (Appendix Table S1). During the same period, the YLLs rates for women aged 45–49 remained relatively stable [0.02% (95% CI: -0.08–0.12%)], while those for women aged 50–54 showed a decreasing trend [-0.14% (95% CI: -0.24%–-0.05%)] (Appendix Table S2).

In most regions, the prevalence of ovarian cancer among perimenopausal women increased from 1990 to 2019, with the largest rise observed in the Caribbean [45–49 years: 3.97% (95% CI: 3.72–4.22%); 50–54 years: 4.11% (95% CI: 3.85–4.37%)] (Appendix Table S1). Over the same period, YLLs rates also increased in most regions, with the Caribbean showing the largest increase [45–49 years: 3.14% (95% CI: 2.91–3.37%); 50–54 years: 3.34% (95% CI: 3.11–3.58%)]. In contrast, a few high-income regions experienced declining YLLs rates, with Australasia showing the steepest decrease [45–49 years: -2.27% (95% CI: -2.43%–-2.12%); 50–54 years: -1.93% (95% CI: -2.23%–-1.63%)] (Appendix Table S2).

In 2019, Monaco reported the highest prevalence of ovarian cancer among perimenopausal women (per 100,000 women) [45–49 years: 204.86 (95% CI: 124.87–314.80); 50–54 years: 229.71 (95% CI: 144.73–336.74)], while Niger had the lowest [45–49 years: 12.90 (95% CI: 6.21–22.18); 50–54 years: 18.60 (95% CI: 9.41–34.09)] (Appendix Table S4). The age-standardized prevalence rates of ovarian cancer for women aged 45–54 in 2019 were presented on the map (Appendix Figure S2). From 1990 to 2019, the percentage change in prevalence varied by country, with 45–49-year-old women in 168 countries and 50–54-year-old women in 168 countries showing an upward trend (Appendix Table S4).

#### Uterine cancer

From 1990 to 2019, the global prevalence of uterine cancer among perimenopausal women showed an increasing trend [45–49 years: 0.99% (95% CI: 0.44–1.55%); 50–54 years: 0.64% (95% CI: 0.37–0.92%)] (Appendix Table S1). In contrast, global YLLs rates for uterine cancer declined during the same period [45–49 years: -1.15% (95% CI: -1.98%–-0.32%); 50–54 years: -1.30% (95% CI: -1.43%–-1.17%)] (Appendix Table S1).

Between 1990 and 2019, the prevalence of uterine cancer among perimenopausal women increased in nearly all regions. The largest increase for women aged 45–49 was observed in High-income Asia Pacific [2.63% (95% CI: 2.12–3.15%)], while for women aged 50–54, the highest rise occurred in South Asia [2.87% (95% CI: 2.06–3.69%)] (Appendix Table S1). Over the same period, YLLs rates declined across most regions, with East Asia experiencing the largest decrease [45–49 years: -2.62% (95% CI: -3.84%–-1.39%); 50–54 years: -2.88% (95% CI: -3.32%–-2.43%)] (Appendix Table S2).

In 2019, the highest prevalence of uterine cancer (per 100,000 women) among perimenopausal women was recorded in the Northern Mariana Islands [45–49 years: 530.71 (95% CI: 291.00–840.02); 50–54 years: 882.93 (95% CI: 514.87–1,425.70)], while the lowest prevalence was observed in Nigeria [45–49 years: 6.45 (95% CI: 3.73–12.36); 50–54 years: 15.07 (95% CI: 8.38–30.87)] (Appendix Table S5). The age-standardized prevalence rates of uterine cancer for women aged 45–54 in 2019 were illustrated on the map (Appendix Figure S3). From 1990 to 2019, prevalence trends varied across countries, with 154 countries showing an upward trend for women aged 45–49 and 159 countries for women aged 50–54 (Appendix Table S5).

### Association with the SDI

#### Cervical cancer

At the regional level, there was a general negative correlation between the age-standardized prevalence rate of perimenopausal cervical cancer and SDI. In regions with medium to low SDI levels, the trend of age-standardized prevalence rates was relatively stable, whereas, in regions with moderate SDI, high-middle SDI, and high SDI, the decreasing trend in prevalence rates was more pronounced, indicating that the prevalence of cervical cancer was lower in areas with higher levels of socio-economic development. Eastern Sub-Saharan Africa, Central Sub-Saharan Africa, Andean Latin America, the Caribbean, and Southern Latin America all had prevalence rates higher than the mean from 1990 to 2019. In contrast, South Asia, North Africa, and the Middle East, among other regions, had prevalence rates below the mean throughout the entire measurement period (Appendix Figure S4). At the country-level, countries such as the Solomon Islands, Botswana, and Northern Mariana lslands had disease prevalence rates significantly above the mean, while Egypt, Palestine, Kuwait, and other countries had age-standardized cervical cancer prevalence rates in perimenopausal women far below the mean (Appendix Figure S5).

#### Ovarian cancer

At the regional level, there was a positive correlation between the age-standardized prevalence rate of cervical cancer in perimenopausal women and SDI, with a turning point generally observed in high-middle SDI regions. The age-standardized prevalence rates of cervical cancer in perimenopausal women showed a decreasing trend in high-middle SDI and high SDI regions but remained higher than in other SDI-level regions. This suggests that regions with a higher level of socio-economic development had higher ovarian cancer prevalence rates. Eastern Sub-Saharan Africa, Southeast Asia, Central Europe, and Eastern Europe all had prevalence rates above the mean from 1990 to 2019. In contrast, Central Sub-Saharan Africa, South Africa, the Middle East, Bangladesh, Southern Sub-Saharan Africa, and Australia had burdens below the mean throughout the entire measurement period (Appendix Figure S6). Countries such as Monaco, Seychelles, and Croatia had age-standardized ovarian cancer prevalence rates in perimenopausal women significantly above the mean, while Chad, the Central African Republic, DR Congo, and other countries had rates far below the mean (Appendix Figure S7).

#### Uterine cancer

At the regional level, there was a positive correlation between the age-standardized prevalence rate of uterine cancer in perimenopausal women and SDI. In regions with high-middle SDI and high SDI, the trend of age-standardized prevalence rates showed a relatively slow increase, indicating that regions with a higher level of socio-economic development also had higher uterine cancer prevalence rates. Oceania, the Caribbean, Central Asia, Central Europe, and Eastern Europe all had prevalence rates higher than expected from 1990 to 2019. In contrast, six countries including South Asia, North Africa, the Middle East, and Southern Sub-Saharan Africa had prevalence rates below the mean throughout the entire measurement period (Appendix Figure S8). Countries such as the Northern Mariana Islands, Georgia, and Latvia had age-standardized uterine cancer prevalence rates in perimenopausal women significantly above the mean, while Nigeria, Malawi, Togo, and other countries had prevalence rates far below the mean (Appendix Figure S9).

### Cross-country inequality analysis

**Table 1:** Health inequalities associated with SDI in the prevalence of cervical, ovarian, and uterine cancers

Diseases	Health inequality metrics			
	Slope index of inequality		Concentration index	
	1990	2019	1990	2019
**Cervical cancer**	-100.98	-170.17	-0.3012	-0.2956
**Ovarian cancer**	75.99	70.66	0.1604	0.0371
**Uterine cancer**	176.51	226.01	0.0954	0.1099

**Fig. 1:**
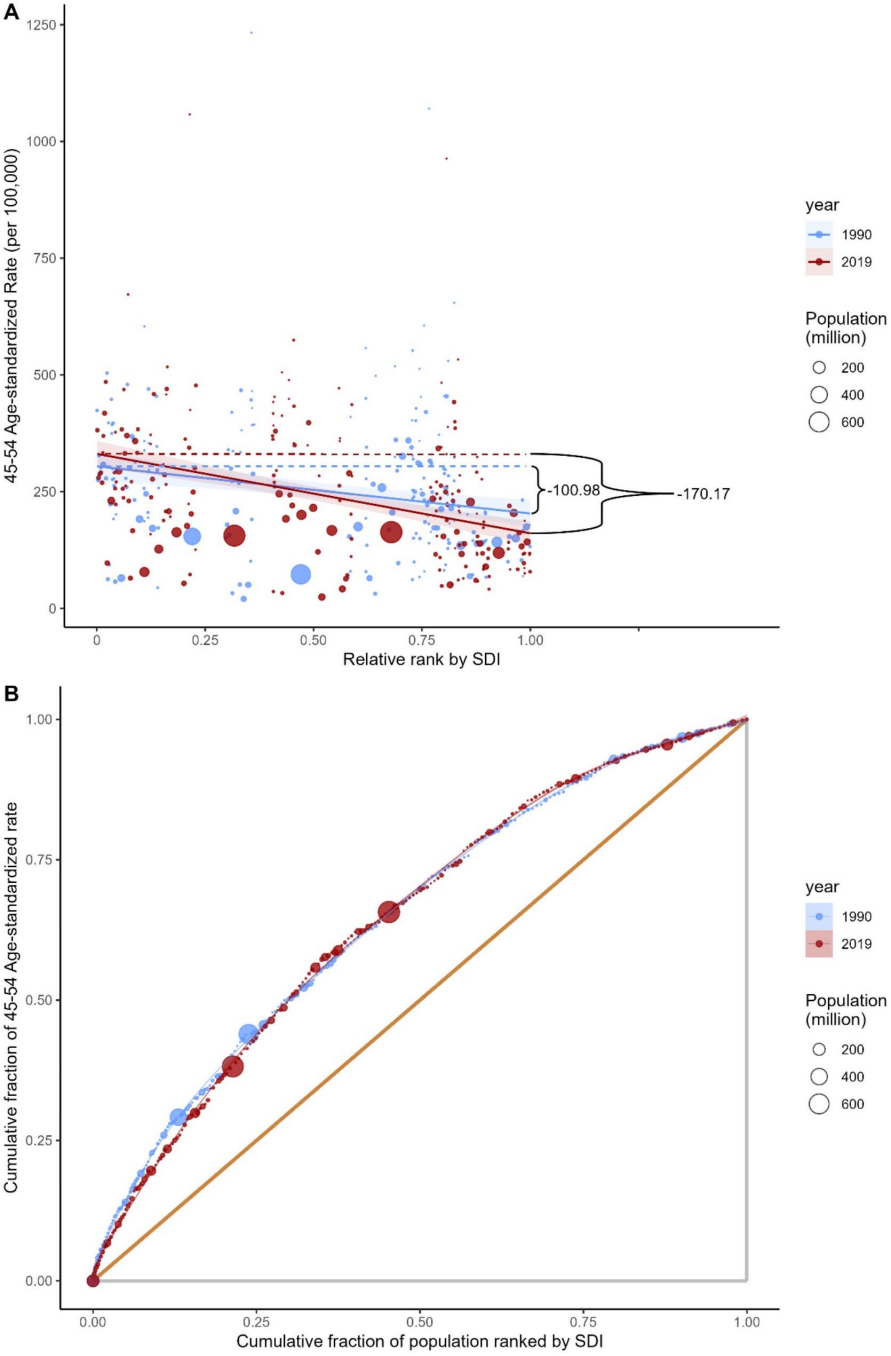
Health inequality regression curve **(A)** and concentration curve **(B)** for global cervical cancer prevalence rates among perimenopausal women from 1990 to 2019

Significant absolute and relative inequalities related to SDI were detected in the burden of gynecological cancers among perimenopausal women. Specifically, concerning cervical cancer health inequalities, higher prevalence rates were disproportionately concentrated in countries with lower SDI. The inequality slope index was − 100.98 in 1990, indicating that compared to countries with the highest SDI in 1990, the prevalence rate exceeded 100.98 (per 100,000) in countries with the lowest SDI, a discrepancy that further widened to 170.17 by 2019 (Table 1). Meanwhile, the concentration index slightly decreased from 1990 to 2019 (Fig. 1).

**Fig. 2:**
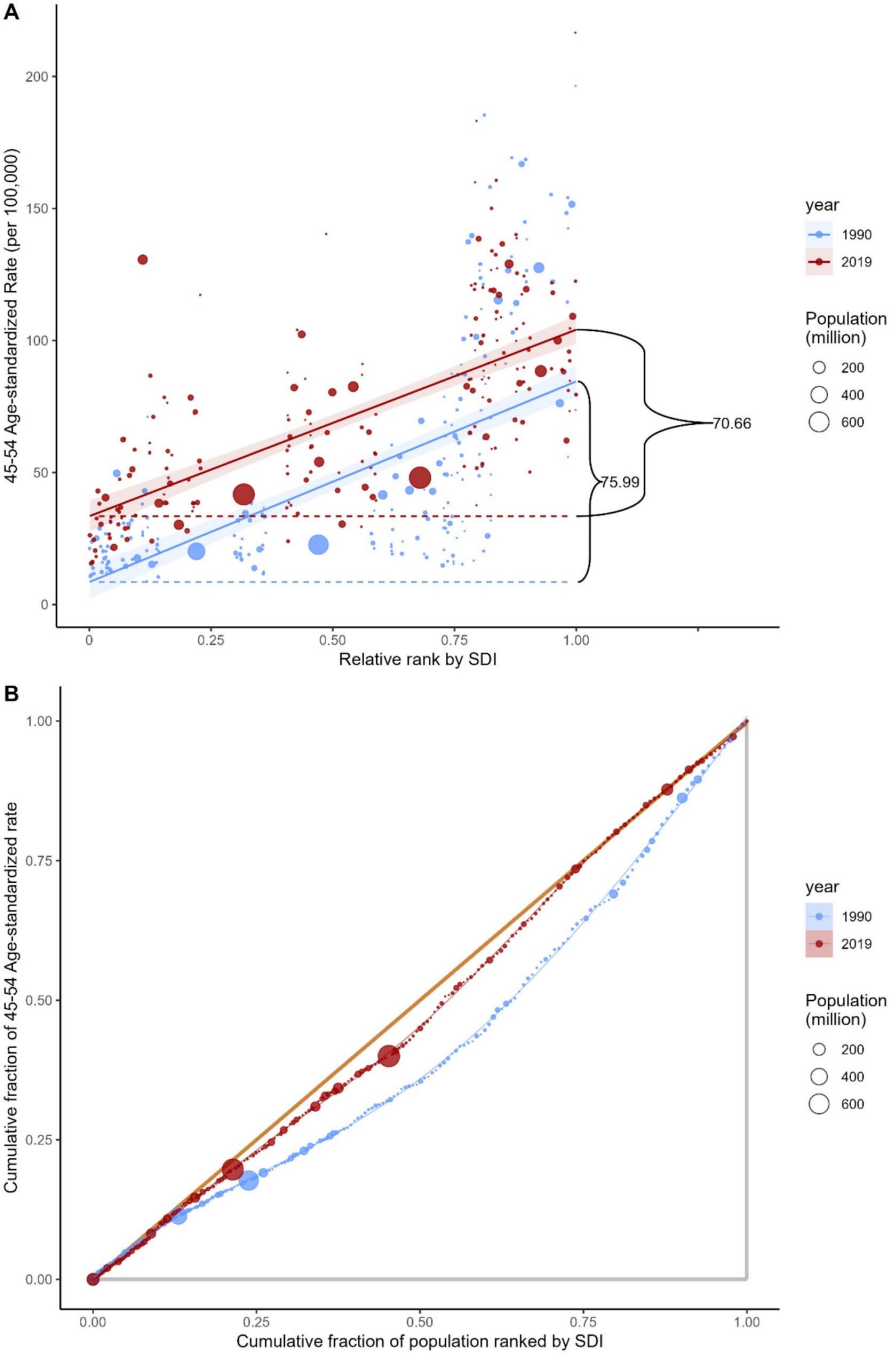
Health inequality regression curve **(A)** and concentration curve **(B)** for global ovarian cancer prevalence rates among perimenopausal women from 1990 to 2019

Regarding ovarian cancer health inequalities, higher prevalence rates were disproportionately concentrated in countries with higher SDI. The inequality slope index was 75.99 in 1990, signifying that compared to countries with the lowest SDI in 1990, the prevalence rate surpassed 75.99 (per 100,000) in countries with the highest SDI, with this gap narrowing to 70.66 by 2019 (Table 1). Concurrently, the concentration index showed a decreasing trend from 1990 to 2019 (Fig. 2).

**Fig. 3:**
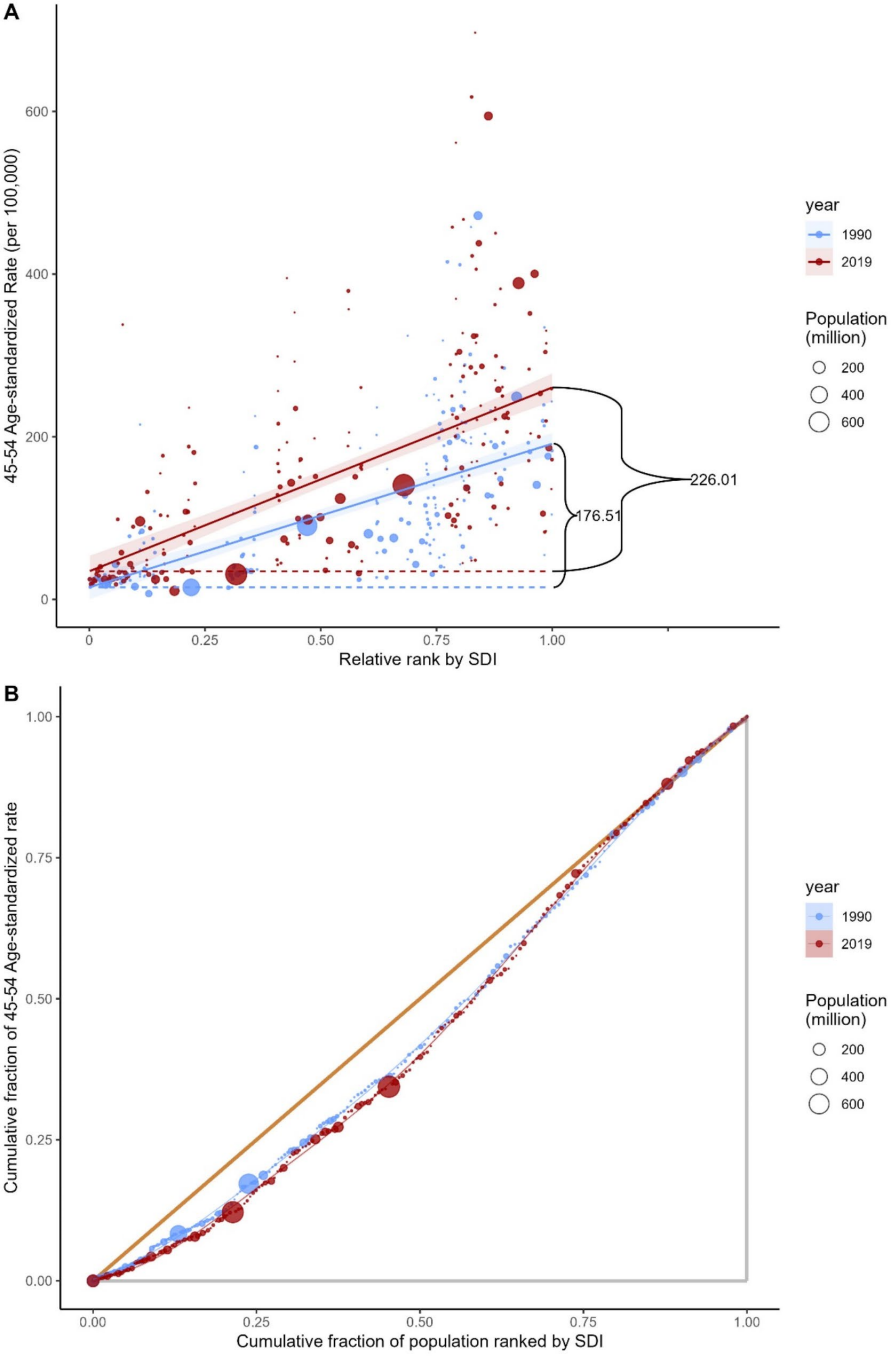
Health inequality regression curve **(A)** and concentration curve **(B)** for global uterine cancer prevalence rates among perimenopausal women from 1990 to 2019

For uterine cancer health inequalities, higher prevalence rates were disproportionately concentrated in countries with higher SDI. The inequality slope index was 176.51 in 1990, indicating that compared to countries with the lowest SDI in 1990, the prevalence rate exceeded 176.51 (per 100,000) in countries with the highest SDI, a discrepancy that further expanded to 226.01 by 2019 (Table 1). The concentration index showed a slight increase from 1990 to 2019 (Fig. 3).

### Frontier analysis

**Fig. 4:**
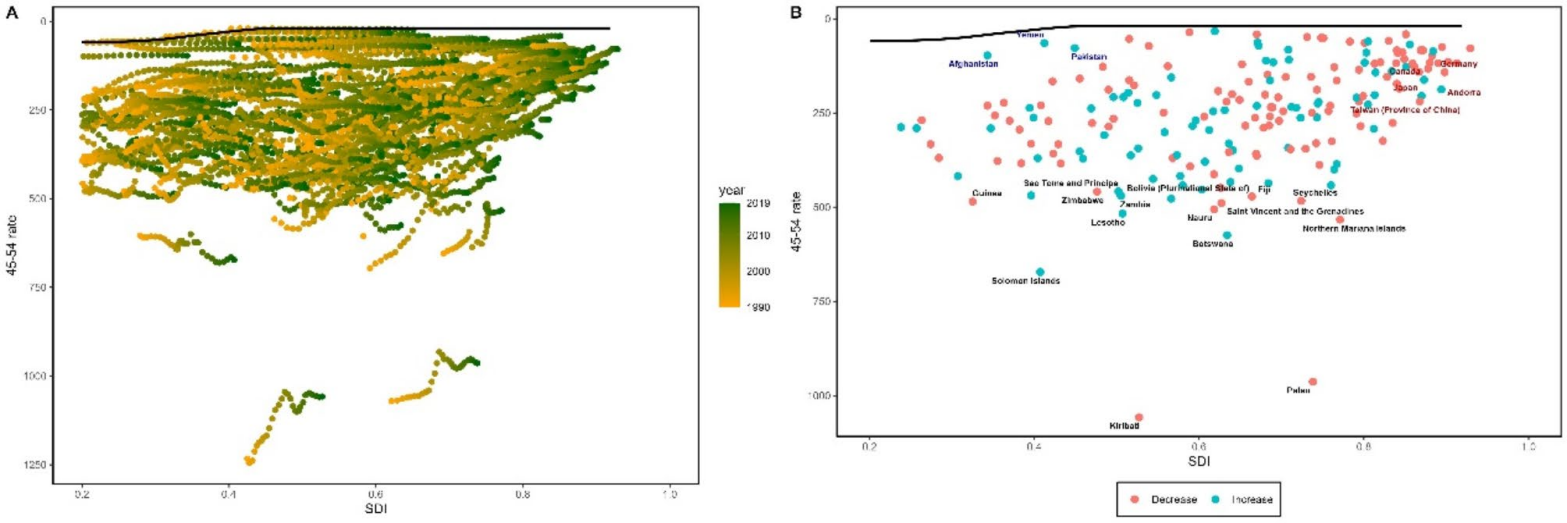
**(a)** Frontier analysis of age-standardized cervical cancer prevalence rates among perimenopausal women based on the Socio-Demographic Index (SDI) and years 1990 to 2019. The color range represents the years from 1990 (gray) to 2019 (green). The frontier is indicated by a solid black line. **(b)** Frontier analysis of age-standardized cervical cancer prevalence rates among perimenopausal women in 2019 based on SDI. The frontier is represented by a solid black line; countries and regions are shown as points. The top 15 countries with the greatest effective difference (the largest gap in cervical cancer prevalence rates among perimenopausal women farthest from the frontier) are highlighted in black; examples of low SDI (< 0.5) and low effective difference countries at the frontier are marked in blue; high SDI (> 0.85) and countries and regions with relatively higher effective differences are highlighted in red. Red points indicate an increase in age-standardized cervical cancer prevalence rates among perimenopausal women from 1990 to 2019; blue points indicate a decrease in age-standardized cervical cancer prevalence rates among perimenopausal women from 1990 to 2019

**Fig. 5:**
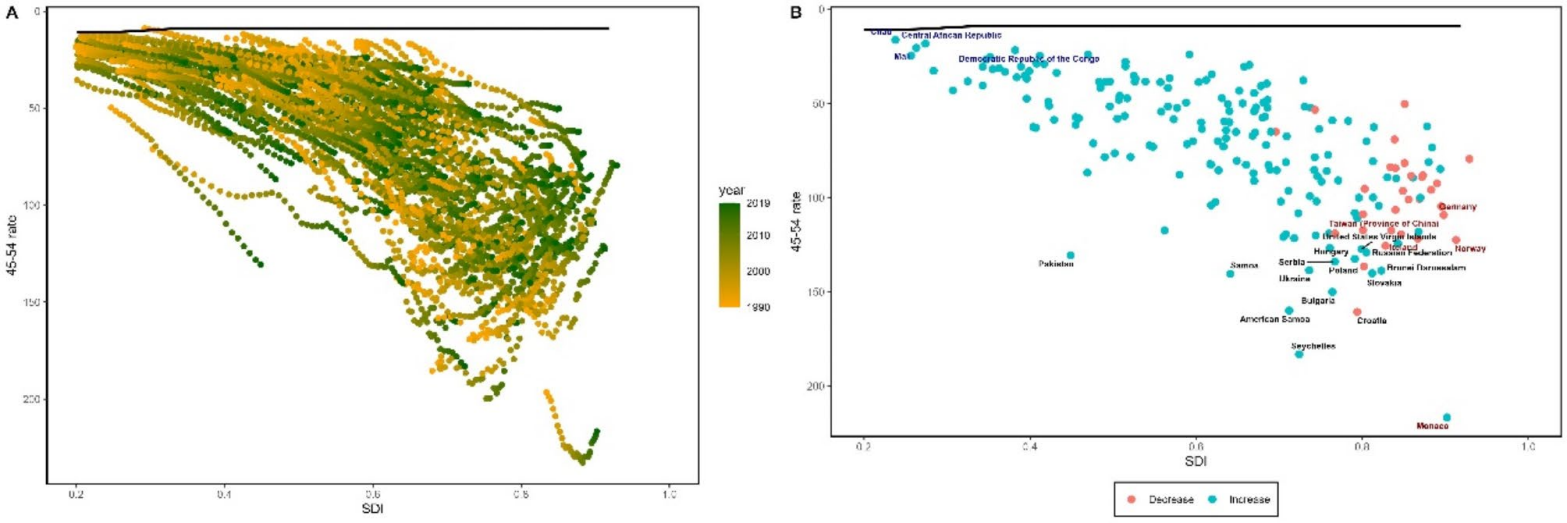
**(a)** Frontier analysis of age-standardized ovarian cancer prevalence rates among perimenopausal women based on the Socio-Demographic Index (SDI) and years 1990 to 2019. The color range represents the years from 1990 (gray) to 2019 (green). The frontier is indicated by a solid black line. **(b)** Frontier analysis of age-standardized ovarian cancer prevalence rates among perimenopausal women in 2019 based on SDI. The frontier is represented by a solid black line; countries and regions are shown as points. The top 15 countries with the greatest effective difference (the largest gap in ovarian cancer prevalence rates among perimenopausal women farthest from the frontier) are highlighted in black; examples of low SDI (< 0.5) and low effective difference countries at the frontier are marked in blue; high SDI (> 0.85) and countries and regions with relatively higher effective differences are highlighted in red. Red points indicate an increase in age-standardized ovarian cancer prevalence rates among perimenopausal women from 1990 to 2019; blue points indicate a decrease in age-standardized ovarian cancer prevalence rates among perimenopausal women from 1990 to 2019

**Fig. 6:**
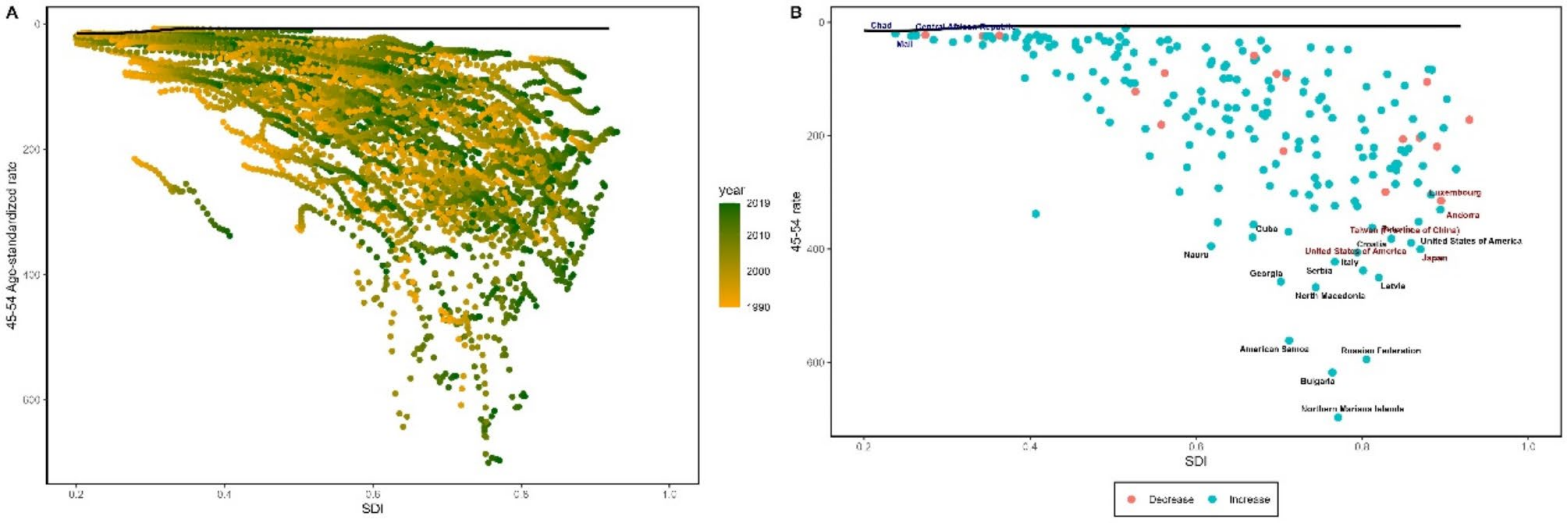
**(a)** Frontier analysis of age-standardized uterine cancer prevalence rates among perimenopausal women based on the Socio-Demographic Index (SDI) and years 1990 to 2019. The color range represents the years from 1990 (gray) to 2019 (green). The frontier is indicated by a solid black line. **(b)** Frontier analysis of age-standardized uterine cancer prevalence rates among perimenopausal women in 2019 based on SDI. The frontier is represented by a solid black line; countries and regions are shown as points. The top 15 countries with the greatest effective difference (the largest gap in uterine cancer prevalence rates among perimenopausal women farthest from the frontier) are highlighted in black; examples of low SDI (< 0.5) and low effective difference countries at the frontier are marked in blue; high SDI (> 0.85) and countries and regions with relatively higher effective differences are highlighted in red. Red points indicate an increase in age-standardized uterine cancer prevalence rates among perimenopausal women from 1990 to 2019; blue points indicate a decrease in age-standardized uterine cancer prevalence rates among perimenopausal women from 1990 to 2019

To better understand the potential improvements in the prevalence rates of female gynecological cancers achievable under varying levels of country development, we conducted a frontier analysis based on age-standardized prevalence rates and SDI from 1990 to 2019 (Figs. 4a and 5a, and 6a). The frontier line demarcated countries and regions with the lowest prevalence rates (best performers) while considering their SDI. The distance from the frontier line, termed effective difference, represented the gap between the observed prevalence rates in countries and the potentially achievable prevalence rates; this gap could be narrowed or eliminated based on each country’s socio-demographic resources. Effective differences for each country and region relative to the boundary were calculated using 2019 data and SDI. Overall, effective differences tended to be small given a certain SDI and exhibited minimal variation with increasing SDI.

In terms of age-standardized prevalence rates of cervical cancer among perimenopausal women, the top 10 countries with the highest effective differences compared to the boundary (effective difference range: 1037.92-457.69) included Kiribati, Palau, Solomon Islands, Botswana, Northern Mariana Islands, Lesotho, Nauru, Saint Vincent and the Grenadines, Seychelles, and Bolivia; these countries had higher prevalence rates of cervical cancer among perimenopausal women than other countries with similar population resources. The top 10 countries with the lowest prevalence rates were positioned within the development spectrum, resulting in the smallest actual differences (range: 12.92–38.27), including the Syrian Arab Republic, Palestine, Niger, Kuwait, Iran, Jordan, Turkey, Bahrain, Sudan, and Qatar (Fig. 4b and Appendix Table S6).

In perimenopausal women with ovarian cancer, the top 10 countries with the highest effective differences compared to the boundary (effective difference range: 207.78-127.39) included Monaco, Seychelles, Croatia, American Samoa, Bulgaria, Samoa, Slovakia, Brunei Darussalam, Ukraine, and Poland; these countries had higher prevalence rates of ovarian cancer among perimenopausal women than other countries with similar socio-demographic resources. The top 10 countries with the lowest prevalence rates were positioned within the development spectrum, resulting in the smallest actual differences (range: 4.59–15.68), including Niger, Chad, Central African Republic, Mali, Democratic Republic of the Congo, Burkina Faso, Dominican Republic, Angola, Somalia, and Yemen (Fig. 5b and Appendix Table S7).

In perimenopausal women with uterine cancer, the top 10 countries with the highest effective differences compared to the boundary (effective difference range: 689.38-398.56) included Northern Mariana Islands, Bulgaria, Russian Federation, American Samoa, North Macedonia, Georgia, Latvia, Italy, Serbia, and Croatia; these countries had higher prevalence rates of uterine cancer among perimenopausal women than other countries with similar socio-demographic resources. The countries with the lowest prevalence rates in the top 10 were positioned within the development spectrum, resulting in the smallest actual differences (range: 3.13–16.04), including Nigeria, Niger, Chad, Central African Republic, Mali, Somalia, Burkina Faso, Malawi, Cote d’Ivoire, and South Sudan (Fig. 6b and Appendix Table S8).

## Discussion

In this study, we reported the prevalence and YLLs rates and trends of cervical, ovarian, and uterine cancers in perimenopausal women. Approximately 1.68 million perimenopausal women globally were estimated to have had these gynecological cancers in 2019. From a global perspective, between 1990 and 2019, the prevalence rates of these three gynecological cancers showed an increasing trend, while YLLs rates exhibited a decreasing trend. This may be related to factors such as the improvement in treatment levels for gynecological cancers and the extension of overall survival rates for patients [9, 27–29]. Disparities in the burden of these three gynecological cancers in perimenopausal women existed based on levels of social development and geographical locations.

Among the three types of cancer, cervical cancer was identified as the most prominent example of cancer-related health inequalities. In countries with a low SDI, women were nearly four times more likely to develop cervical cancer compared to women in high SDI countries [30]. The findings indicated that the age-standardized incidence rate of cervical cancer in postmenopausal women was negatively correlated with SDI, which was consistent with other research [31]. Additionally, cervical cancer incidence rates demonstrated a declining trend in high-middle and high SDI regions. Nearly all cases of cervical cancer (99%) were associated with high-risk human papillomavirus (HPV) infection [32]. Tools for primary prevention (prophylactic HPV vaccination) and secondary prevention (screening and treatment of cervical precancerous lesions using validated HPV testing) were demonstrated to be effective [31]. The implementation of these measures may have contributed to the decline in global YLLs rates for cervical cancer among perimenopausal women. In high-income countries, cervical cancer incidence and mortality decreased by more than half over the past three decades following the introduction of formal screening programs [33]. For example, in this study, Australasia showed a significant decline in cervical cancer rates. Australia, one of the first countries to introduce a national HPV vaccination program, was part of this region. It was projected that by 2028, the incidence would decline to fewer than 4 new cases per 100,000 women [34]. In contrast, in low- and middle-income countries, the lack of formal screening programs resulted in persistently high incidence rates and an increasing number of cases [33].

Our study found that in most regions, the YLLs rates of ovarian cancer in perimenopausal women showed an increasing trend. This trend may be related to late-stage diagnoses in the majority of ovarian cancer cases, leading to poor prognosis [35]. The age-standardized prevalence rate of ovarian cancer in perimenopausal women was generally positively correlated with the SDI level of the region. Research has confirmed that factors such as limited breastfeeding, nulliparity or infertility, and obesity are risk factors for ovarian cancer [36–40]. In Europe, a region belonging to high-middle SDI areas, overall breastfeeding rates remain low [41], which could contribute to the persistently high prevalence of ovarian cancer in European regions. Additionally, quantifying transnational inequalities related to SDI in the prevalence of perimenopausal ovarian cancer revealed that absolute inequalities in ovarian cancer among perimenopausal women have decreased compared to 1990 but still persist, predominantly affecting health equality in economically developed countries. Although industrialization cannot be fully equated with high socio-demographic development, ovarian cancer is the type of cancer with the lowest survival rate in industrialized countries [42]. In 2009, the International Agency for Research on Cancer (IARC) concluded that there is sufficient evidence to link asbestos exposure to ovarian cancer [43], highlighting significant healthcare needs for this disease even in higher-income nations due to the exposure to toxic substances associated with industrialization and urbanization in high SDI regions and countries, coupled with the stealthy onset of ovarian cancer [23]. It is essential to note regional health inequalities; in our study, the YLLs rates of perimenopausal ovarian cancer showed an increasing trend in most regions from 1990 to 2019, while several regions with higher prevalence rates and higher socio-economic levels exhibited a decreasing trend in YLLs. Therefore, addressing perimenopausal ovarian cancer requires both reducing prevalence rates in high socio-economic levels regions and ensuring better outcomes in other regions. Overall, while there has been a slight alleviation in the inequality phenomenon related to SDI in ovarian cancer prevalence among perimenopausal women globally, it remains a significant issue. This suggests that over the past 30 years, adjustments have been made in the prevention, management, and treatment of ovarian cancer with the improvement in socio-demographic development levels, but further sustained investments in the treatment and prevention of this disease are necessary.

The research findings indicated a positive correlation between the age-standardized prevalence rate of uterine cancer in perimenopausal women and the SDI level of the region. An analysis of transnational inequality in uterine cancer among perimenopausal women revealed that countries with higher SDI bore a disproportionate burden of uterine cancer. It is generally believed that individuals living in countries and regions with higher SDI were more likely to access and enjoy higher quality health and medical services, potentially bearing a lesser disease burden. However, in this study, over time, the overall increase in the inequality phenomenon related to the burden of uterine cancer among perimenopausal women across countries associated with SDI was significant. This suggested that over the past 30 years, despite the improvement in socio-demographic development levels, investments in the prevention, management, and treatment of uterine cancer in perimenopausal women might have been inadequate.

The study proposed that the unusual correlation observed between the age-standardized prevalence rate of uterine cancer and socio-demographic development levels might be related to women’s lifestyles. A report indicated that Asian women with uterine cancer had a survival advantage over white women; however, due to environmental exposures and high-fat diets, uterine cancer is increasing significantly among Asian immigrant women born in the United States [44]. There was evidence that high-income countries tended to have higher obesity rates [45], and due to the association between high body mass index (BMI) and increased risk of uterine cancer [18], women in these countries were at greater risk of developing uterine cancer. Additionally, as women entered the perimenopausal stage, the risk of obesity often increased [8], which further contributed to the high prevalence of uterine cancer in countries with a higher SDI.

The Frontier analysis of this study indicated that there was still significant room for improvement in the control of reproductive cancer prevalence among perimenopausal women across countries with different SDI levels. Some high SDI countries showed lagging performance in the treatment and prevention of uterine and ovarian cancer prevalence rates. Moving forward, conducting disease control efforts, identifying factors hindering the progress of disease control in economically advanced countries, and addressing this knowledge gap might help provide crucial information for alleviating the burden of perimenopausal gynecological cancers.

Based on existing gender disparities and unequal access to treatment, women with cancer in low- and middle-income countries face greater vulnerabilities [46]. Although cervical and uterine cancers are the most common and highly treatable [47, 48] cancers among women, disparities in healthcare accessibility prevent many women from receiving timely treatment [49]. Moreover, gender norms and socio-cultural factors exacerbate these challenges. In many cultures, women are expected to prioritize family needs over their own health, often at the expense of their well-being, while men are frequently excluded from familial caregiving responsibilities [50].In socially conservative environments, particularly among low-income families, women often lack financial autonomy and must rely on men to cover medical expenses or transportation costs to healthcare facilities [50]. This reliance often results in their healthcare needs being overlooked or delayed. Reducing gender inequality can improve women’s living conditions and health status.

The perimenopause is associated with an increased risk of depression [16]. According to Sustainable Development Goal 3, by 2030, a one-third reduction in premature mortality from non-communicable diseases through prevention and treatment is targeted, promoting mental health and well-being [51]. Therefore, strengthening psychological counseling for perimenopausal women affected groups is essential to help them cope with emotional changes, stress, anxiety, and other mental health challenges. Simultaneously, developing mental health policies tailored to perimenopausal women involves advancing social protection systems and healthcare institutions to offer more mental health services, including attention to and intervention measures for menopause-related issues.

Since ovarian cancer is not easily detected in its early stages [52], it is more likely to result in a poorer prognosis than uterine or cervical cancer [53]. The government could enhance early ovarian cancer detection through free or low-cost gynecological screenings and regular check-ups. Subsidizing advanced therapies, expanding insurance coverage, and supporting technological innovations could potentially improve treatment accessibility and effectiveness. Additionally, healthcare institutions are encouraged to provide genetic counseling services to assist individuals with a family history of ovarian cancer, as well as specific populations such as Ashkenazi Jewish women [54], in understanding their risk of developing the disease and to offer necessary monitoring and intervention measures. Information about early signs of ovarian cancer, dietary habits, lifestyle, and prevention methods was disseminated to the public through various media channels to raise awareness.

In conclusion, medical resources for cervical cancer screening and prevention need to be directed towards countries with lower economic levels. According to the “Global Strategy to Eliminate Cervical Cancer,” targets were set to achieve by 2030: 70% of women undergoing high-quality screening methods between the ages of 35 and 45, and 90% of women diagnosed with cervical diseases receiving treatment [32]. In higher-income regions, attention should be paid to the impact of perimenopausal female obesity on uterine and ovarian cancers, with tailored policies and resource allocation.

This study also had some limitations. Firstly, cases from underdeveloped countries in the GBD might have been underestimated due to poor healthcare performance, potentially leading to underdiagnosis, missing records, and other issues. Secondly, the research findings based on the GBD heavily relied on modeled data since a variety of sophisticated statistical models were used to account for the varying quality of raw data collected from different countries. Additionally, the GBD lacked data for the 45–54 age group, necessitating manual calculation which only yielded point estimates without providing 95% confidence interval estimates. Finally, although ACOG defined the age range of perimenopausal women as 45–54 years, there were still some individual variations.

## Conclusions

This study found that the prevalence of gynecological cancers among perimenopausal women globally has been increasing over the past 30 years, resulting in a significant disease burden and posing a severe challenge to global public health. Firstly, from 1990 to 2019, health inequalities in cervical and uterine cancer among perimenopausal women have widened, and there are regional health disparities in the burden of ovarian cancer. Moreover, the frontier analysis of this study indicates that there is still considerable room for improvement in controlling the prevalence of gynecological cancers among perimenopausal women in countries with different levels of SDI. To address these challenges, it is essential to develop region-specific strategies and adapt interventions accordingly.

## Electronic supplementary material

Below is the link to the electronic supplementary material.

Supplementary Material 1

Supplementary Material 2

## Data Availability

The data that support the findings of our study are openly available in Global Health Data Exchange, at https://gbd2019.healthdata.org/gbd-results/. Further information is available from the corresponding author upon request.

## Abbreviations

YLL: Years of life lost
SDI: Socio-Demographic Index
WHO: World Health Organization
ACOG: The American College of Obstetricians and Gynecologists
