# Supplementary material for: Health inequality and improvement gap in the prevalence of gynecological cancers among perimenopausal women globally, 1990–2019
Source: BMC Public Health. 2025 Feb 12;25:590. doi: 10.1186/s12889-025-21807-3 (PMC11823132; doi:10.1186/s12889-025-21807-3)
Supplement: Supplementary file 2 — Supplementary Material 2 [file 12889_2025_21807_MOESM2_ESM.docx]

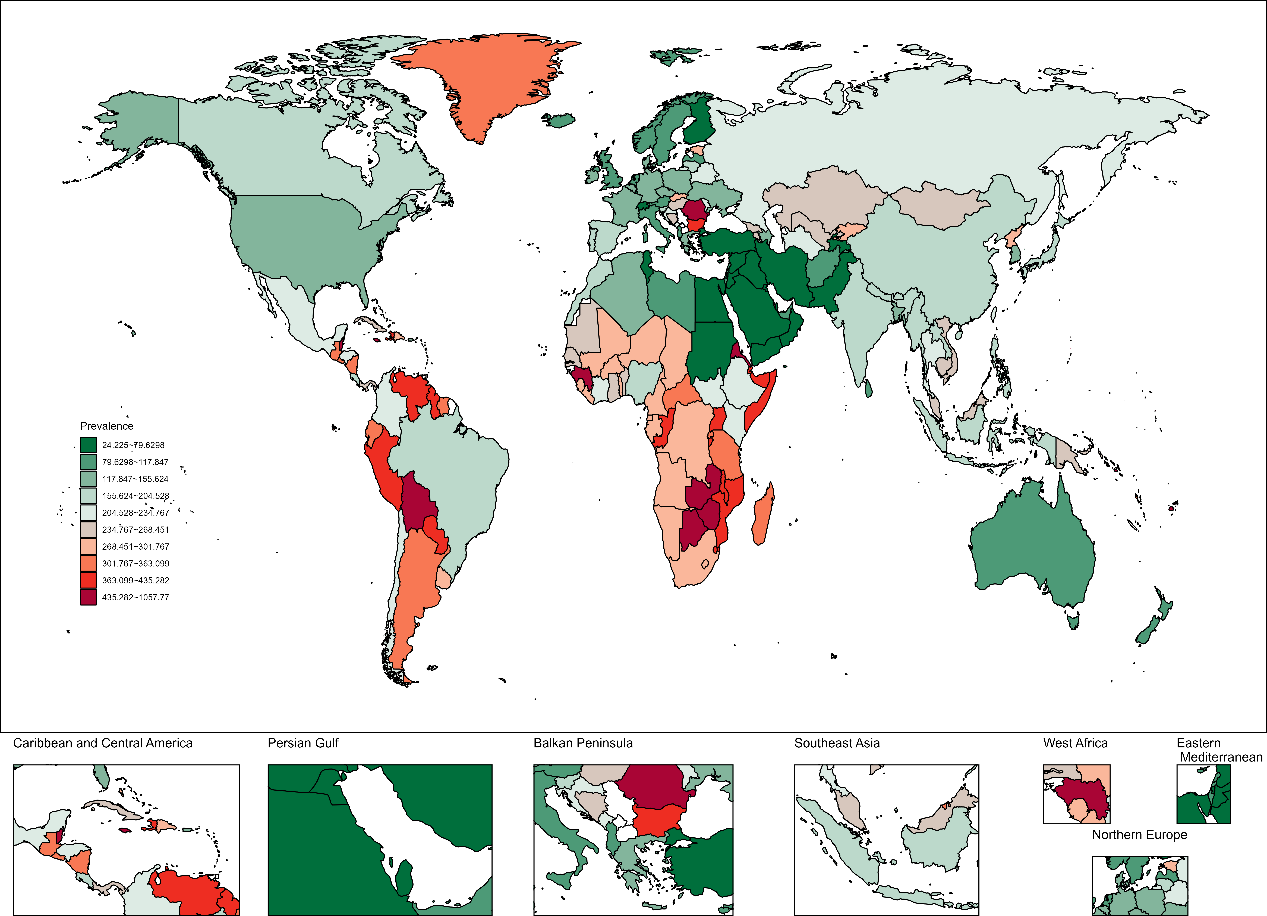


Figure S1 Cervical cancer prevalence rates among perimenopausal women by country/region in 2019.


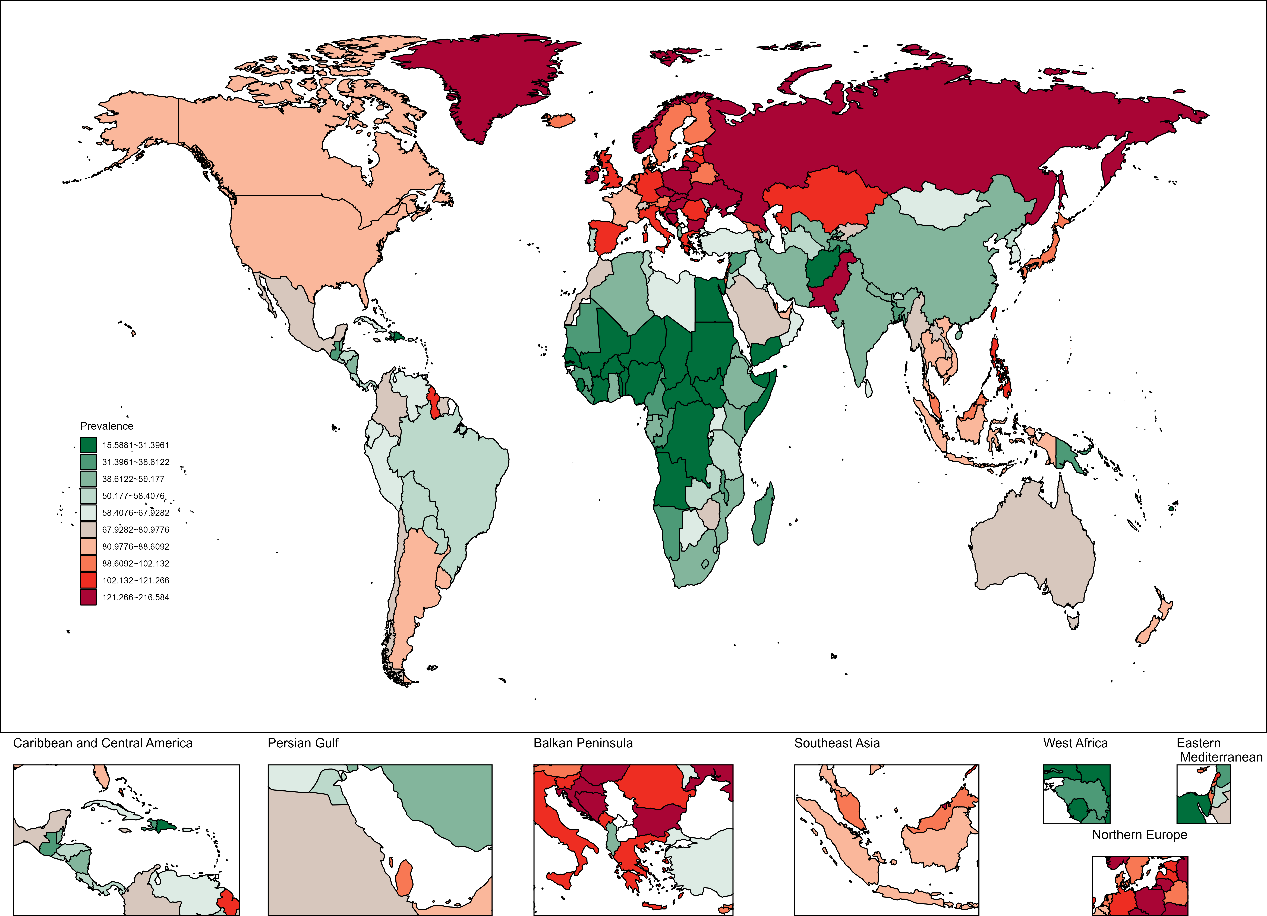


Figure S2 Ovarian cancer prevalence rates among perimenopausal women by country/region in 2019.


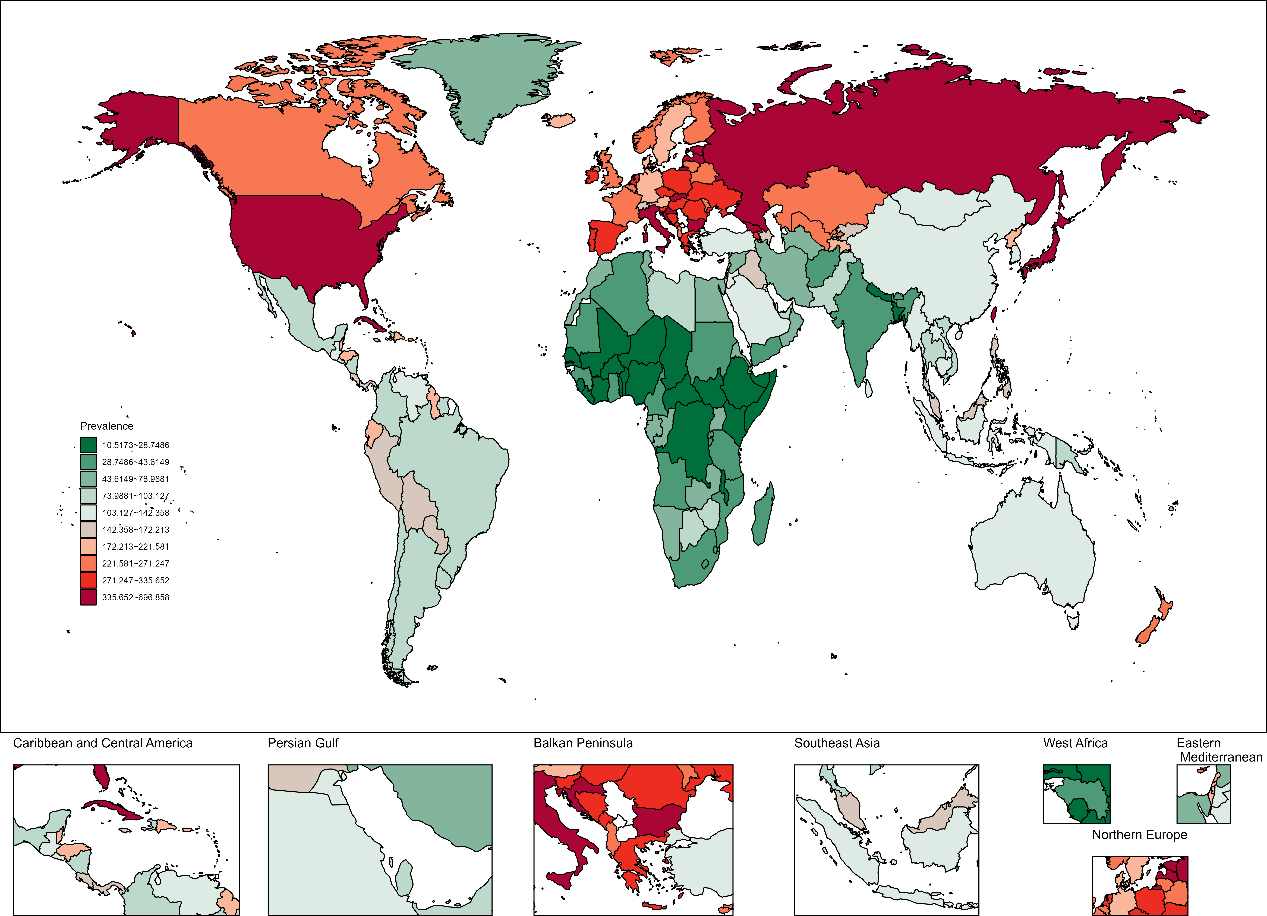


Figure S3 Uterine cancer prevalence rates among perimenopausal women by country/region in 2019.

**
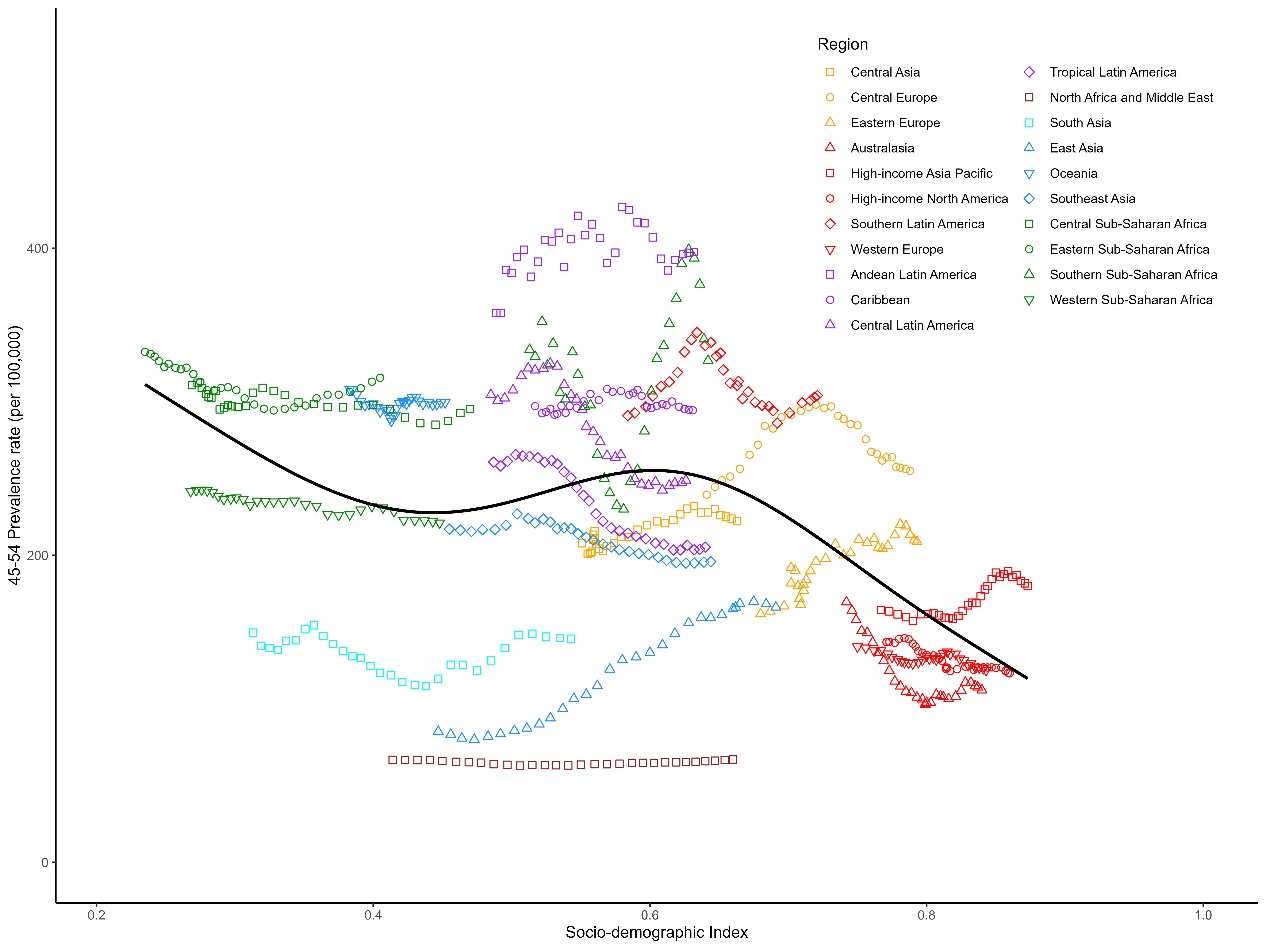
**

Figure S4 Age-standardized cervical cancer prevalence rates among perimenopausal women in 21 regions globally from 1990 to 2019, based on the SDI; Expected values based on the SDI and disease rates are displayed as a black line. Each GBD region is represented by 30 data points showing the age-standardized cervical cancer prevalence rates observed in that region from 1990 to 2019.

*SDI: Socio-Demographic Index


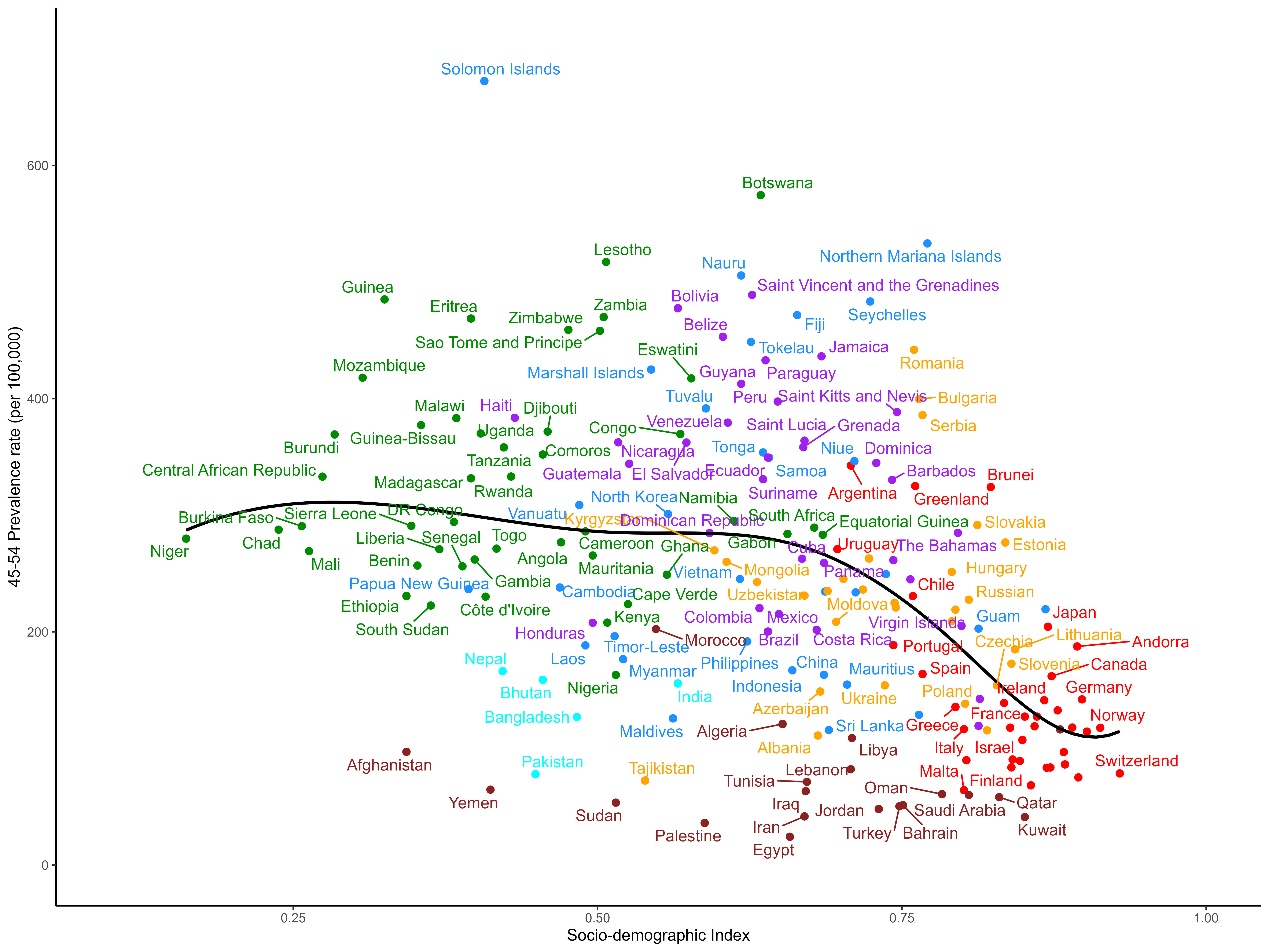


Figure S5: Age-standardized cervical cancer prevalence rates among perimenopausal women in 204 countries and regions in 2019, based on the SDI; Expected values based on the SDI and disease rates are displayed as a black line. Each data point shows the age-standardized cervical cancer prevalence rates observed in each country in 2019

*SDI: Socio-Demographic Index

**
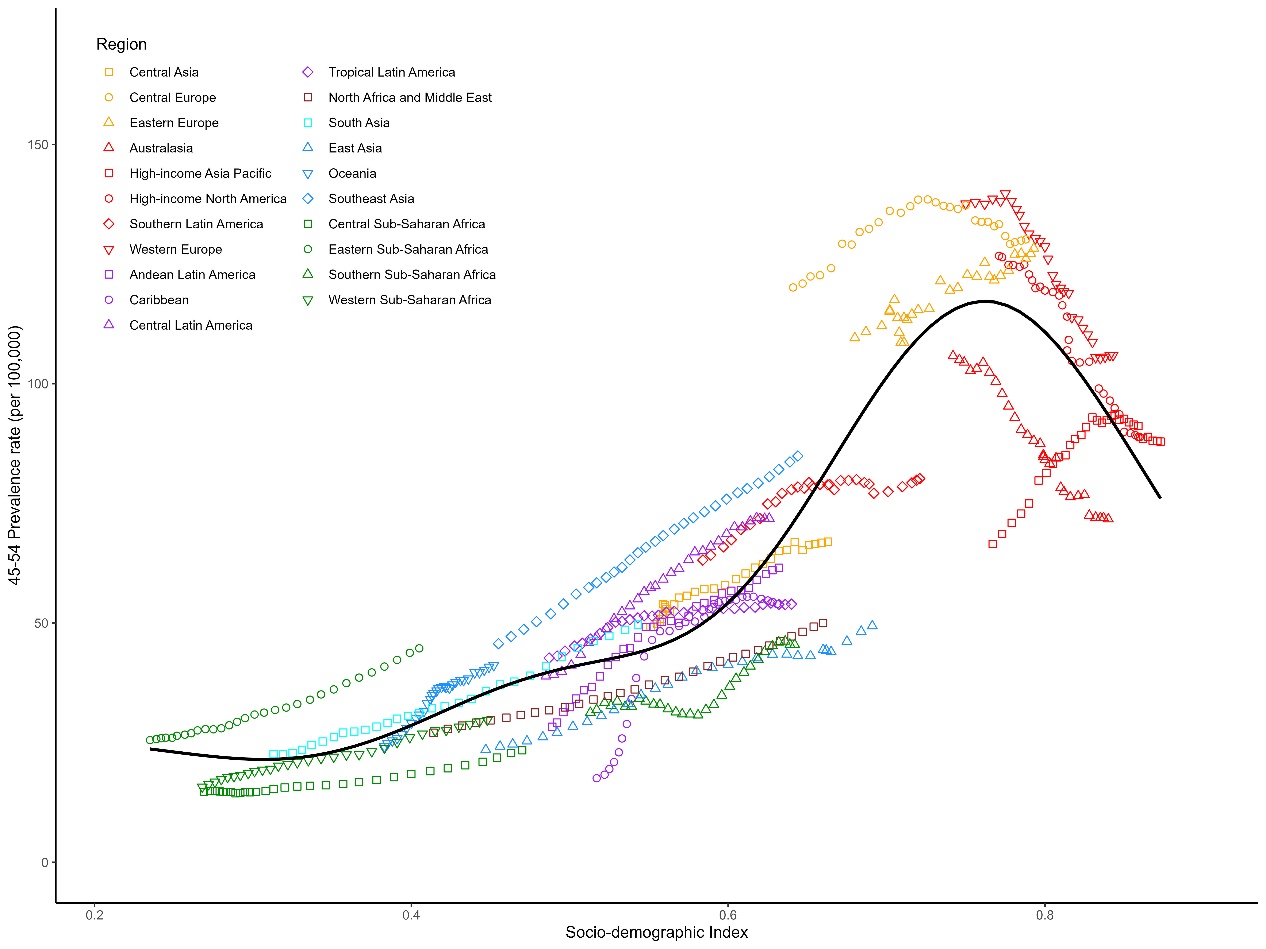
**

Figure S6 Age-standardized ovarian cancer prevalence rates among perimenopausal women in 21 regions globally from 1990 to 2019, based on the SDI; Expected values based on the SDI and disease rates are displayed as a black line. Each GBD region is represented by 30 data points showing the age-standardized ovarian cancer prevalence rates observed in that region from 1990 to 2019.

*SDI: Socio-Demographic Index


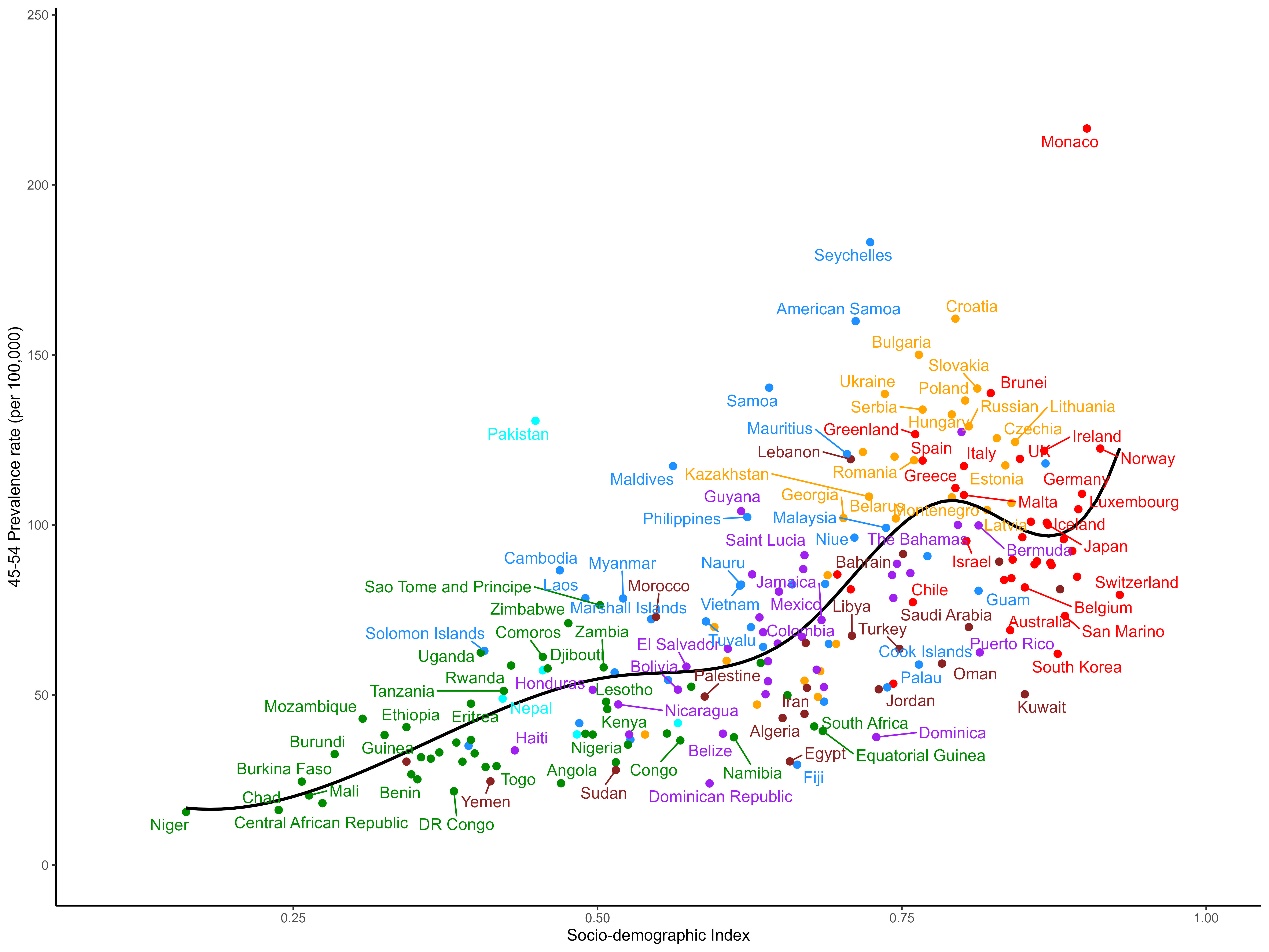


Figure S7: Age-standardized ovarian cancer prevalence rates among perimenopausal women in 204 countries and regions in 2019, based on the SDI; Expected values based on the SDI and disease rates are displayed as a black line. Each data point shows the age-standardized ovarian cancer prevalence rates observed in each country in 2019.

*SDI: Socio-Demographic Index

**
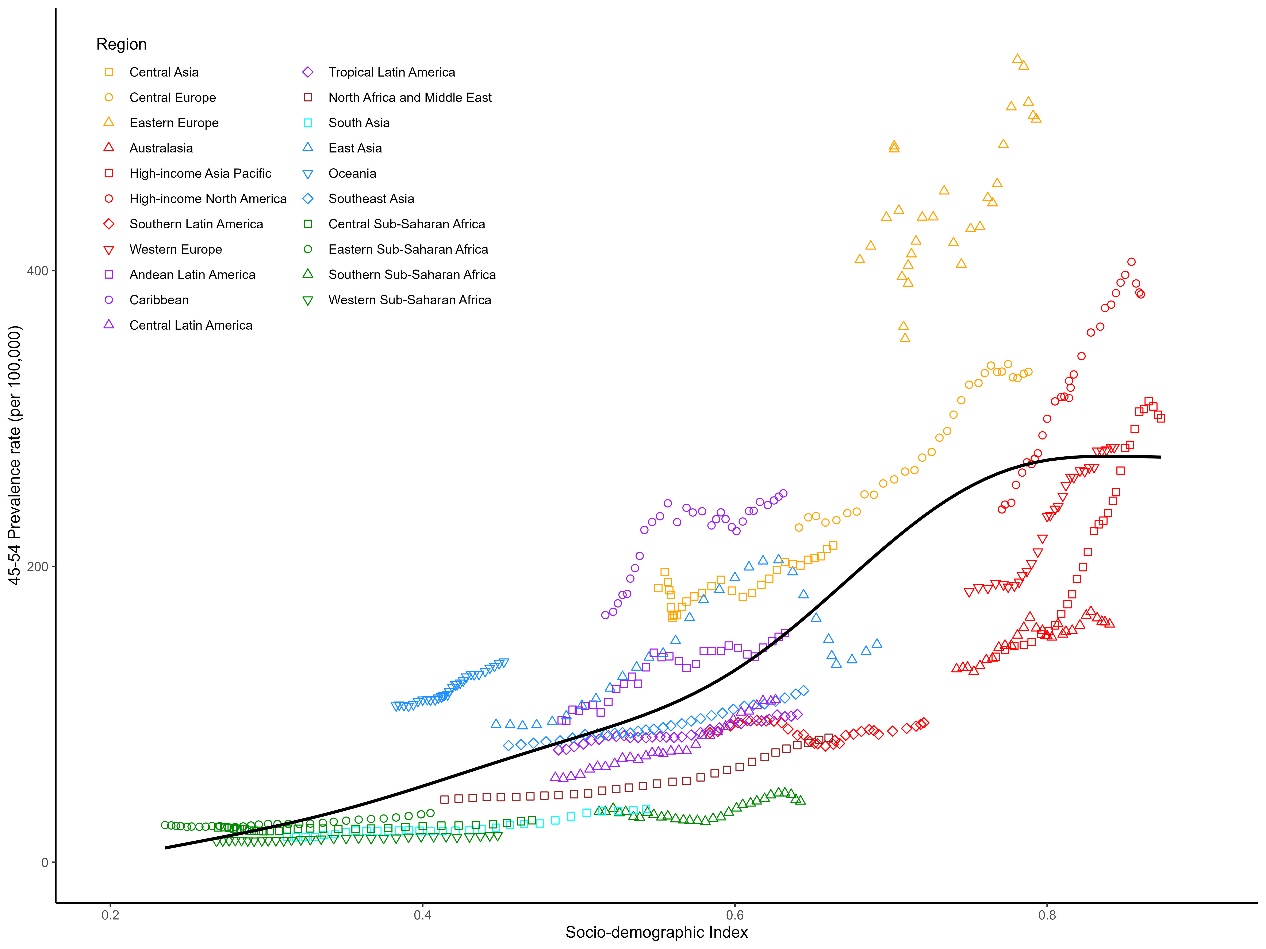
**

Figure S8 Age-standardized uterine cancer prevalence rates among perimenopausal women in 21 regions globally from 1990 to 2019, based on the SDI; Expected values based on the SDI and disease rates are displayed as a black line. Each GBD region is represented by 30 data points showing the age-standardized uterine cancer prevalence rates observed in that region from 1990 to 2019.

*SDI: Socio-Demographic Index


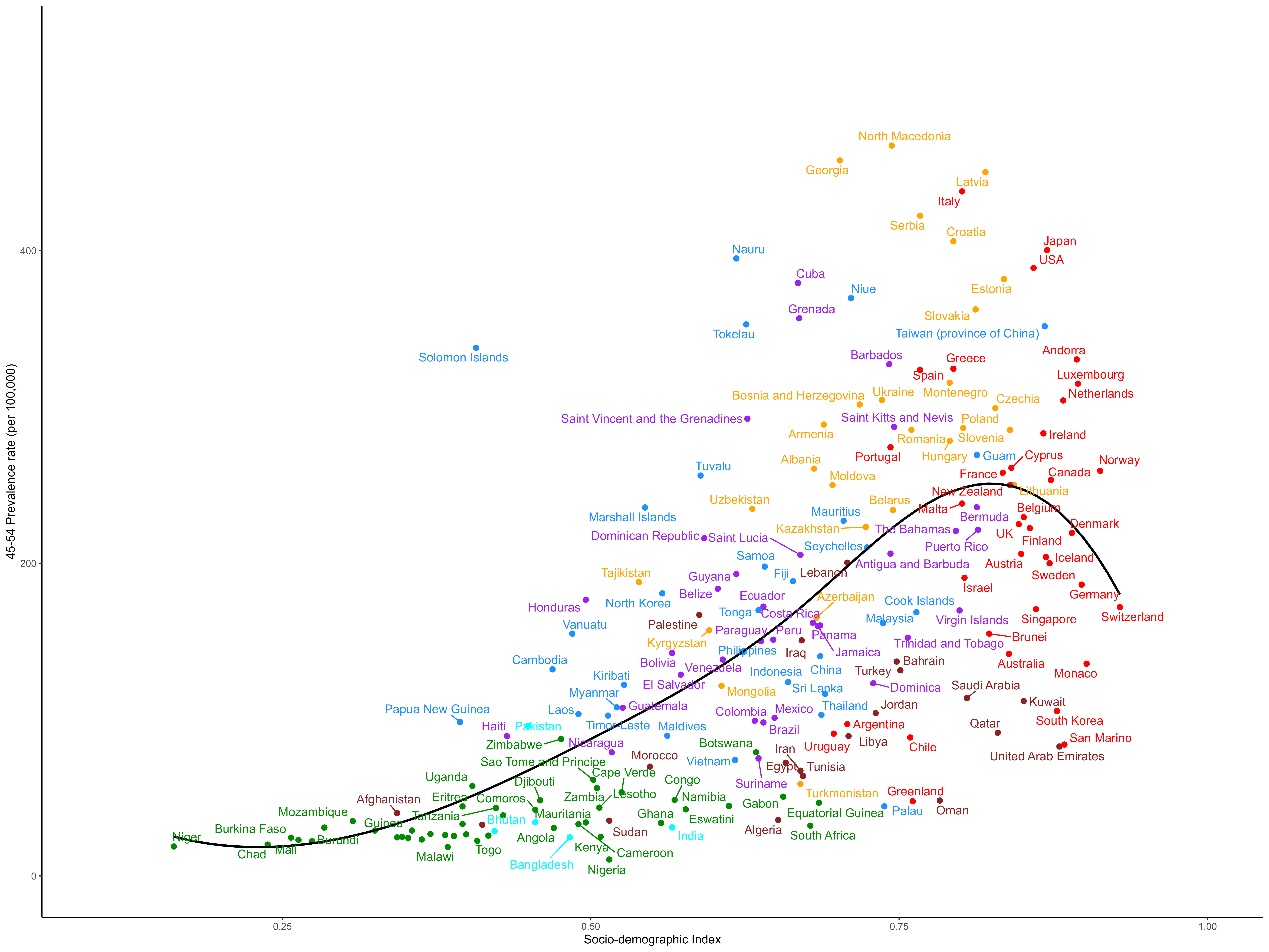


Figure S9 Age-standardized uterine cancer prevalence rates among perimenopausal women in 204 countries and regions in 2019, based on the SDI; Expected values based on the SDI and disease rates are displayed as a black line. Each data point shows the age-standardized uterine cancer prevalence rates observed in each country in 2019.

*SDI: Socio-Demographic Index
